# Genetic Structure and Molecular Variability of *Cucumber mosaic virus* Isolates in the United States

**DOI:** 10.1371/journal.pone.0096582

**Published:** 2014-05-06

**Authors:** Shahideh Nouri, Rafael Arevalo, Bryce W. Falk, Russell L. Groves

**Affiliations:** 1 Department of Plant Pathology, University of Wisconsin, Madison, Wisconsin, United States of America; 2 Department of Botany, University of Wisconsin, Madison, Wisconsin, United States of America; 3 Department of Plant Pathology, University of California Davis, Davis, California, United States of America; 4 Department of Entomology, University of Wisconsin, Madison, Wisconsin, United States of America; Institute of Infectious Disease and Molecular Medicine, South Africa

## Abstract

*Cucumber mosaic virus* (CMV) has a worldwide distribution and the widest host range of any known plant virus. From 2000 to 2012, epidemics of CMV severely affected the production of snap bean (*Phaseulos vulgaris* L.) in the Midwest and Northeastern United States. Virus diversity leading to emergence of new strains is often considered a significant factor in virus epidemics. In addition to epidemics, new disease phenotypes arising from genetic exchanges or mutation can compromise effectiveness of plant disease management strategies. Here, we captured a snapshot of genetic variation of 32 CMV isolates collected from different regions of the U.S including new field as well as historic isolates. Nucleotide diversity (π) was low for U.S. CMV isolates. Sequence and phylogenetic analyses revealed that CMV subgroup I is predominant in the US and further showed that the CMV population is a mixture of subgroups IA and IB. Furthermore, phylogenetic analysis suggests likely reassortment between subgroups IA and IB within five CMV isolates. Based on phylogenetic and computational analysis, recombination between subgroups I and II as well as IA and IB in RNA 3 was detected. This is the first report of recombination between CMV subgroups I and II. Neutrality tests illustrated that negative selection was the major force operating upon the CMV genome, although some positively selected sites were detected for all encoded proteins. Together, these data suggest that different regions of the CMV genome are under different evolutionary constraints. These results also delineate composition of the CMV population in the US, and further suggest that recombination and reassortment among strain subgroups does occur but at a low frequency, and point towards CMV genomic regions that differ in types of selection pressure.

## Introduction

*Cucumber mosaic virus* (CMV), the type species of the genus *Cucumovirus* in the family *Bromoviridae*, is one of the most widespread plant viruses causing disease in various crop and non-crop plants in the United States and worldwide. The CMV host range includes over 1,000 plant species comprised of both crop and non-crop plant species [1], [2]. CMV is transmitted by over 75 species of aphids in a non-persistent manner [2]. The genome of CMV contains three, positive-sense, single-stranded RNAs packaged in separate particles [2], [3]. Virus particles also contain two subgenomic RNAs [1], [3], [4]. RNA 1 and 2 encode the 1a and 2a proteins, respectively, which constitute two subunits of the virus replicase complex [5]. RNA 2 also encodes the 2b protein which is a multifunctional protein involved in host-specific, long-distance movement, symptom induction, and as a virulence determinant by suppressing gene silencing [4], [6]–[8]. Moreover, a recent study has demonstrated that the 2b gene determines the selection of inter-viral recombination [9]. The CMV RNA 3 encodes two proteins, 3a, a cell-to-cell movement protein (MP) [8] and 3b or the capsid protein (CP); this latter protein being translated from a sub-genomic RNA 4 [10]. CP is involved in cell-to-cell movement, virion assembly and aphid-mediated transmission [11]–[15].

CMV strains have been classified into two main subgroups designated as subgroups I and II based on serology [2], [16], nucleic acid hybridization [17], RT-PCR followed by RFLP [18] and nucleotide sequence identity [2], [19]. These two subgroups show 75% nucleotide identity [19] and subgroup I is more heterogeneous than subgroup II [1]. Further analysis of the CP gene and the 5′ non-translated region (NTR) of RNA 3 has led to further division of subgroup I into IA and IB with 92–95% nucleotide identity between these two subgroups [19], [20]. Phylogenetic analysis of some CMV strains showed that the estimated trees for various open reading frames (ORFs) located on the different RNAs are not congruent and do not completely support the subgrouping from CP ORF analysis. This indicates that different RNAs may have independent evolutionary histories [19].

The subgroups are not evenly distributed across agricultural regions. Subgroups IA and II have a worldwide distribution, while subgroup IB is reported to be principally restricted to Asia [20]. RNA viruses can undergo rapid genetic change, and random mutation, recombination and reassortment are the most common sources of RNA virus evolution and variability [21]–[24]. Reassortment among RNAs within all three CMV subgroups has been reported [19], [25]–[27]. Moreover, recombination in the 5′ and 3′ NTR, between ORFs 3a and 3b [20], [25], [28]–[30] and in natural populations of CMV containing satellite RNA [31], has been shown to be additional sources of variability in the virus population. Hence, CMV is a heterogenic species with significant variation among isolates. This provides the virus with the ability to rapidly evolve in unique environments with shifting selection pressures [19], [27], [32]–[37]. Studies focusing on this genetic diversity and sources of variation in the viral populations are important to better understand evolutionary mechanisms that generate variation.

CMV has been endemic in many parts of the United States for decades. However, during the 2000s, a series of virus epidemics occurred that affected processing snap bean (*Phaseolus vulgaris* L.) in agriculturally important regions of the upper Midwest and Northeast US [38]–[41]. The appearance of such plant virus epidemics can be partially explained by emergence of novel variants in virus populations. However, there is currently insufficient molecular-based phylogenetic information to describe CMV structure and subgroup distribution in the United States. In the current study, we provide a snapshot of genetic variation of CMV isolates in the US and made comparisons among these US isolates to include new isolates, reference isolates and historically curated isolates. Moreover, we determined the sources of genetic variation and discuss potential evolutionary mechanisms acting upon domestic CMV isolates included in the investigation. In a practical context, this information can be very useful towards development of more comprehensive virus disease control strategies.

## Materials and Methods

### Virus isolates and propagation

CMV isolates included in this study were obtained from two principal sources. First, a subset of isolates (Table 1) was obtained from historical research collections where flash frozen, or freeze-dried plant tissues were provided. We do not possess detailed information describing the sequence of host plants (or number of passages), through which these isolates had previously been maintained. Another subset of isolates included in this investigation (Table 1) were obtained from field- collected and symptomatic plants which had tested positive for CMV infection by serology. Taken together, a discrete set of 32 isolates were included in the study to represent a range of geographic locations throughout the US as well as a range of several collection years. Specifically, we attempted to represent isolates from among discrete regions in the US including the western region (Arizona, California, Oregon, and Hawaii), the upper Midwestern region (Wisconsin), the midsouth region (Arkansas, Kentucky) and the eastern regions (New York, Maryland, New Jersey) of the country. In addition, isolates were obtained from 10 different host plant species, including collections in succulent snap beans from New York and Wisconsin where recent outbreaks of CMV have emerged [38]. Finally, four CMV isolates (PV544NJU04, PV30MDH85, PV243AZM77 and PV29WIC76) were obtained from the American Type Culture Collection (ATCC) (Manassas, VA, USA) (Table 1) as dried plant tissue and included in the current study as reference control standards. Prior to characterization, all dried/frozen virus isolates were first mechanically inoculated into small sugar pumpkin (*Cucurbita pepo* L. cv. ‘Small Sugar’) plants. For inoculation, leaves were ground in 0.1 M potassium phosphate buffer (0.1 M potassium phosphate, pH 7.4, containing 0.05% Na_2_SO_3_) and recipient leaves were mechanically sap-inoculated. Inoculated plants were maintained in an insect-proof greenhouse at 21–25 °C and 16:8 (L:D) photoperiod for 4 weeks post-inoculation. Leaves from symptomatic plants were then vacuum-dried and stored at −20 °C prior to analysis.

**Table 1:** CMV isolates collected in the United States and included in the phylogenetic analysis.

Isolate	Geographic origin	Host	Year of collection	Isolate	Geographic origin	Host	Date collected
AORU93	Oregon	Unknown	1993	NNYS09*	New York	Snap Bean	2009
BORU93	Oregon	Unknown	1993	ONYS09*	New York	Snap Bean	2009
FORU93	Oregon	Unknown	1993	HWH10*	Hawaii	*Commelina diffusa*	2010
HORU94	Oregon	*Primula* sp.	1994	NS3WIS09*	Wisconsin	Snap bean	2009
KYKTT08	Kentucky	Tobacco	2008	TWIS08*	Wisconsin	Snap bean	2008
CaNYU90	New York	Unknown	1990	WWIS07*	Wisconsin	Snap bean	2007
CENYC90	New York	Cucumber	1990	MirrorWIS07*	Wisconsin	Snap bean	2007
PNYC90	New York	Cucumber	1990	3ARS50	Arkansas	Spinach	1950
V154NYT85	New York	Tomato	1985	113CAP90	California	Pepper	1990
V85NYT80	New York	Tomato	1980	116CAP90	California	Pepper	1990
PV243AZM77	Arizona	Mungbean	1977	160ECAP90	California	Pepper	1990
PV29WIC76	Wisconsin	Cucumber	1976	MDCAP93	California	Pepper	1993
PV30MDH85	Maryland	*Commelina nudiflura*	1985	144ICAP90	California	Pepper	1990
PV544NJU04	New Jersey	Unknown	2004	INYS09*	New York	Snap Bean	2009
LNYS09*	New York	Snap Bean	2009	JNYS09*	New York	Snap Bean	2009
MNYS09*	New York	Snap Bean	2009	KNYS09*	New York	Snap Bean	2009

Underlined isolates were obtained from ATCC.

Isolates marked with (*) are field-collected.

### RNA extraction, RT- PCR amplification and Sequencing

Total RNA extraction was performed on all samples using an RNeasy Plant Mini kit (Qiagen, Valencia, CA) according to the manufacturer's instructions. The first strand cDNAs were synthesized in a 20 µl volume of 1× Superscript III reaction buffer (Invitrogen, Carlsbad, CA), containing 0.5 mM dNTPs mix, 5 mM DTT, 40 U RNaseOut, 200 U of SuperScript III Reverse Transcriptase (Invitrogen, Carlsbad, CA), and 20 pmol of a specific reverse primer for each region [27]. Subsequent PCR reactions were conducted in 25 µl volume of 1× GoTaq Flexi DNA Polymerase reaction buffer (Promega, Madison, WI), containing 0.2 mM dNTP mix, 1.1 mM MgCl_2_, 0.75 U of Go Taq Flexi DNA Polymerase (Promega, Madison, WI) and 12.5 mM of each forward and reverse primer [27]. The thermal cycles were as follows: 5 min at 94°C followed by 22 cycles at 94°C for 30s, 54°C (CP, MP, 2a and 3′ NTR) and 51°C (1a and 2b) for 30s, 72°C for 1 minute (80s for 1a) and finished by 72°C for 7 minutes. Gel purifications were performed using a QIAquick gel extraction (Qiagen, Valencia, CA). Purified products for each genomic region/isolate combination were bi-directionally sequenced using a model 377 ABI PRISM DNA sequencer (Perkin-Elmer, Waltham, MA) in the Automated DNA Sequencing Facility of the University of California-Davis. Consensus sequences of each genomic region/isolate combination were obtained using the NTI Vector Advance 11 program (Invitrogen, Carlsbad, CA) and later used for phylogenetic analysis.

### Sequence alignment, phylogenetic analysis and estimation of population genetic parameters

For analyses, reference isolate sequences were included as representatives of CMV subgroups IA, IB and II. All reference sequences were downloaded from GenBank (www.ncbi.nlm.nih.gov/nuccore) (Table S1). Multiple nucleotide sequence alignments were performed using CLUSTALW in MEGA version 5 [42] and alignments were manually adjusted in MacClade 4.08 [43]. Aligned CMV sequences were assessed using DnaSP software version 5.1 [44], to estimate genetic diversity and other population genetic parameters. Appropriate nucleotide substitution models for each partition were determined using the program jModelTest 2 [45], [46], and the models nominated by the Akaike information criterion (AIC), which were applied in each case (TIM2+I+G for 1a; GTR+G for 2a and 2b; SYM+G for CP; GTR+I+G for MP; and TPM3+G for 3′NTR). Bayesian consensus phylogenetic trees were inferred using Mr Bayes 3.2 software [47], [48]. For each analysis, two independent sets of four metropolis-coupled Monte Carlo Markov chains were run with estimated priors for 10,000,000 generations, sampling every 1000 generations, with a burn-in of 25% and chains heated to 0.10.

### Recombination Analysis

To detect possible recombination between different CMV isolates, automatic recombination scans of sequence alignments were carried out using the RDP3 program [49]. In total, 7 recombination detection methods were implemented and included RDP [49], [50], Bootscan [51], GENECONV [52], MaxChi [53], [54], Chimaera [53], Siscan [55] and 3SEQ [56]. The program was run using the default settings plus the Bonferroni corrected P-value cut-off (α = 0.05). Recombination events were considered as significant if four or more methods had a consensus P-value ≤0.01, in addition to phylogenetic evidence of recombination. The results obtained in the recombination analysis by RDP were confirmed using a boot scanning method [51] in the SimPlot program [57]. The window width and the step size were set to 200 and 20 bp, respectively.

### Neutrality tests

To assess selection pressure imposed upon CMV coding regions, non-synonymous (dN, amino-acid altering) and synonymous (dS, silent) substitution rates and their associated ratios (dN/dS =  ω) were estimated for each segment by using the bootstrap method with 500 replicates under the Kumar method [58] in MEGA version 5 [42]. To determine site specific selection pressure in each coding region, three complementary maximum-likelihood methods including single likelihood ancestor counting (SLAC), fixed effects likelihood (FEL), and random effects likelihood (REL) [59], [60] implemented in the Hyphy package (http://www. Datamonkey.org) were applied. To classify a site as positively or negatively selected, the cut-off P-value was selected to be 0.1 for SLAC and FEL. For REL, a Bayes factor of 50 was selected as the cut-off value. The most appropriate nucleotide substitution models were selected for each gene by the software and only selections determined to be significant by at least two methods were considered as positive selections.

## Results

### Genetic diversity of selected CMV isolates

**Figure 1:**
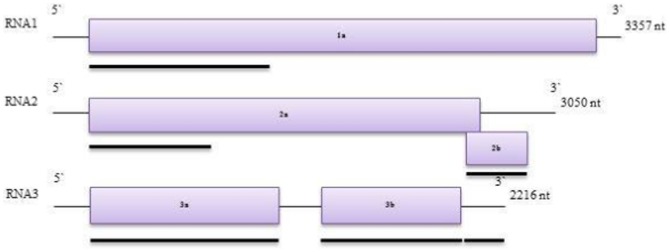
Genome organization of CMV. Black bars indicate the genomic regions analyzed in this study.

A total of thirty-two CMV isolates (Table 1) were included in this study and cDNA fragments representing 6 genomic regions of CMV (Figure 1) were amplified from these CMV isolates with RT-PCR. The six regions generated amplicons of 1098, 653, 378, 848, 678 and 315 bp in length, respectively for each of the viral genomic segments. The number of isolates for each genomic segment amplified with specific primer sets is illustrated in Table 2. Associated population genetic parameters were estimated (Table 2) including π, the average pairwise nucleotide difference per site, and θw, the mutation rate from segregating number, and these estimators were used as two indicators of genetic diversity for each genomic region. Overall, the genetic diversity for U.S. CMV isolates was low with a mean genetic diversity of 0.037. Specifically, the 2b region showed the highest genetic variation among CMV coding regions followed by MP, 2a, CP and 1a considering both genetic variation estimations.

**Table 2:** Population genetic parameters estimated for coding regions of the U.S. CMV isolates using the DnaSP and MEGA programs.

Genomic region	Number of isolates	1S	2η	3π	4 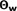	5dS	6dN	7ω (dN/dS)
1a	24	65	66	0.014	0.017	0.037±0.006	0.005±0.002	0.13
2a	27	65	67	0.032	0.030	0.066±0.012	0.020±0.005	0.30
2b	28	76	79	0.058	0.059	0.100±0.021	0.045±0.009	0.45
								
MP	26	117	124	0.036	0.036	0.098±0.012	0.010±0.002	0.10
CP	26	72	76	0.030	0.028	0.088±0.012	0.006±0.002	0.07
3′NTR (RNA3)	28	54	56	0.050	0.046	n/a	n/a	n/a

1 Total number of segregating sites.

2 Total number of mutations.

3 Nucleotide Diversity, average pairwise nucleotide difference per site.

4 Mutation rate estimated from S.

5 The average number of pairwise differences per synonymous site.

6 The average number of pairwise differences per non-synonymous site.

7 dS and dN were estimated by Kumar method.

### Phylogenetic relationship of CMV isolates

**Figure 2:**
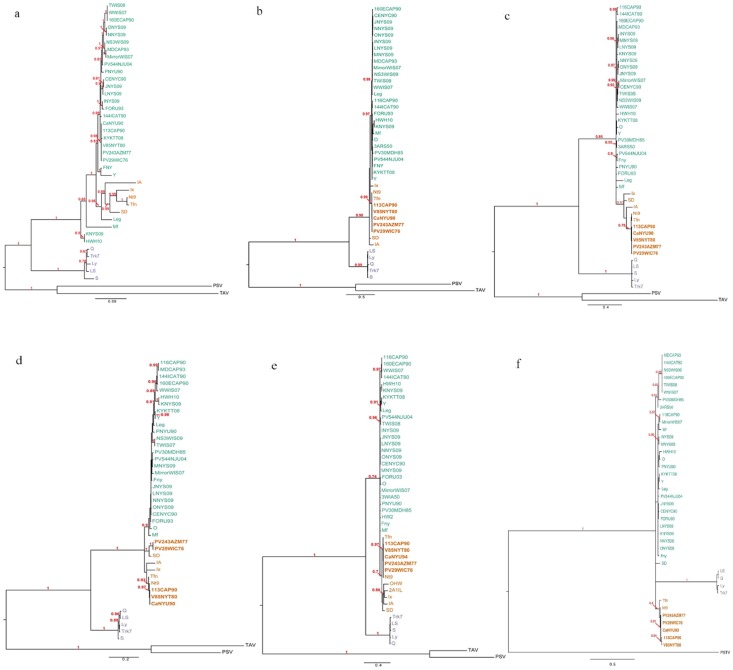
Bayesian phylogenetic trees of CMV isolates based on six genomic regions including field collected and reference isolates. (a) 1a, partial sequence; (b) 2a, partial sequence; (c) 2b, complete sequence; (d) MP, complete sequence; (e) CP, complete sequence; and (f) RNA3 3’NTR complete sequence. Numbers above branches indicate Bayesian inference posterior probability (PP). CMV subgroups are differentiated by colors: IA  =  green, IB  =  orange, II  =  purple. Reassortants have illustrated in bold.

Phylogenetic trees were constructed for U.S. CMV isolates based on partial nucleotide sequences of the 1a and 2a ORFs, and full nucleotide sequences of the 2b, MP, CP ORFs and the 3′NTR of RNA 3 (Figure 2). Fifteen reference sequences obtained from GenBank were included in the phylogenetic analysis (Table S1). For the CP gene tree, three extra reference isolates originated from the U.S. were used (Table S1). Divergence of subgroups I and II, with high supporting values, was observed in all phylogenetic trees. Based on these analyses, subgroup I was the predominant subgroup among U.S. isolates included in this investigation (Figure 2). Two isolates belonging to subgroup II were recognized according to the MP gene tree, but further analyses suggested that these may have been recombinants (details in the recombination analysis section) and were removed from the data set for the remainder of the analyses. The presence of both subgroups IA and IB in the U.S. was confirmed by phylogenetic analyses, and the divergence of these two subgroups was clear in most trees (Figure 2). According to the CP phylogenetic gene tree, subgroup IA and IB isolates were found on separate clades and this separation was well-supported (Figure 2e). U.S. CMV historical isolates included in this study represented a mixture of both subgroup IA and IB, while all new isolates collected in the last decade (e.g. 2002–2007) belonged to subgroup IA (Figure 2e) based on the CP gene tree. These newly collected isolates were sampled from snap bean, with the exception of “HWH10” and “PV544NJU04” isolates, which were collected from other hosts (Table 1). Furthermore, two clades were generated within the subgroup IB. All U.S. subgroup IB isolates examined in this study were clustered together in one clade of the CP gene tree, separately from two other subgroup IB reference isolates (OHW and 2A1IL previously collected from various locations in the U.S.) which were placed in other clade (Figure 2e). These newly identified subgroup IB isolates in the U.S. illustrated a close phylogenetic relationship with the ‘Nt9’ and ‘Tfn’ reference isolates, also determined to be subgroup IB strains and isolated from Taiwan and Italy, respectively, with high supporting values (Figure 2e). The remaining isolates were grouped with the CMV subgroup IA reference isolates and created a single clade called subgroup IA (Figure 2e). This latter subgroup was of limited diversity. An interesting point was that four CMV isolates (‘JNYS09’, ‘LNYS09’, ‘NNYS09’ and ‘ONYS09’) collected in 2009 in New York were clustered with another CMV isolate (CENYC90) collected from the same geographic region but at least 20 years earlier (Figure 2). However, no obvious association between phylogenetic groups and other factors such as geographic location, year and host species was seen according to the CP gene tree.

The topology of the MP phylogenetic gene tree with, more defined branches, was different from the CP gene tree (Figure 2d). Subgroup IB isolates are separated from the rest of subgroup IA isolates and both subgroups illustrated more diversity compared to the CP tree (Figure 2d). U.S. subgroup IB isolates were placed in two separate clades (Figures 2d). Two additional subgroup IB isolates (‘PV29WIC76’ and ‘PV243AZM77’) were separated from the remaining isolates and formed a unique clade which illustrated a close phylogenetic relationship with a reference subgroup IB strain (‘SD’) submitted from China also with the highest posterior probability (Figure 2d). Similar to the CP gene tree, the remainder of subgroup IB isolates formed a cluster with ‘Nt9’ and ‘Tfn’ strains (Figure 2d). Conversely, subgroup IA U.S. isolates formed a large clade with reference isolates from the same subgroup (Figure 2d). Based on the MP gene tree, groups within the subgroup IA are not the same as groups within the same subgroup in the CP gene tree.

Based on the 2a gene tree, subgroup IB clustering followed a similar trend as for the CP gene tree (Figure 2b). On the other hand, two main branches were seen inside of the subgroup IA clade in the 2a gene tree forming two U.S. CMV groups, one with more phylogenetic relationship to the ’Leg‘ isolate obtained from Japan (Figure 2b), and the other group illustrated closer phylogenetic relationship to other subgroup IA reference isolates. The similar pattern of the 2a gene subgroup IB clustering was observed for four identified subgroup IB isolates in the 2b gene tree (Figure 2c). However, topology of the 2b gene tree was different from 2a gene tree (Figures 2b and 2c), suggesting independent evolutionary histories of these two genes originating from the same RNA.

In contrast to the other gene trees, subgroup IA and IB were not clearly separated based on the 2b gene tree (Figure 2c). Further divergence of the U.S. CMV isolates included in the current study into IA and IB was not obvious based on the 1a gene tree, so that all U.S. isolates fell into subgroup IA clade with more branching inside of this specific subgroup (Figure 2a). This indicates an evolutionary history that is quite different from what was seen in other RNAs. It was interesting that the ‘HWH10’ and ‘KNYS09’ isolates formed a unique clade separately from the reminder of isolates (Figure 2a). Although these two isolates formed a separate clade inside the 1a tree, they were still recognized as subgroup IA isolates (Figure 2a).

The topology of the 3′NTR tree was quite different from other phylogenetic trees (Figure 2f). However, as far the other trees, divergence of subgroups IA and IB was consistent here (Figure 2f).

Overall, no clear, significant associations between phylogenetic groups with location (e.g. state or origin of collection), host plant species, or collection year was observed and this was consistent for all phylogenetic trees (Figure 2).

### Reassortment trace

To assess the potential for genetic exchange resulting from reassortment among the representatives of the U.S. CMV population, phylogenetic trees of full nucleotide sequences of 2b, MP, CP, RNA3 3′NTR, as well as partial sequences of 1a and 2a together with additional reference CMV isolates from GenBank (Table S1) were compared. These phylogenetic comparisons suggested at least five obvious reassortants among US isolates included in the current study. Specifically, analysis of the RNA3 (MP, CP and 3′NTR) and RNA2 (2a and 2b) placed the isolates ‘PV243AZM77’, ‘PV29WIC76’, ‘CaNYU90’, ‘V85NYT80’ and ‘113CAP90’ into subgroup IB, whereas they belonged to subgroup IA based on analysis of the RNA1 (1a) segment alone (Figure 2). These data suggested the potential for these five isolates to be the result of natural reassortment between subgroups IA and IB at some point in their history.

### Recombination analysis

Phylogenetic analysis further illustrated evidence of recombination for two CMV isolates included here, ‘BORU93’ and ‘HORU94’. These two isolates were assigned to subgroup II based on the phylogenetic tree of the MP genomic region), but were assigned to subgroup I according to the CP and 3′NTR trees (data not shown). Since these three regions all occur on RNA3, recombination may be a possible explanation for this outcome. To further investigate this phylogenetic signature of recombination, we concatenated nucleotide sequences of MP, CP and the 3′NTR and subsequently evaluated them using the RDP3 package. Here, the RDP3 package scanned the aligned sequences using multiple methods. Six out of seven methods implemented in this package suggested recombination events for these two isolates with highly significant P-values (Tables S2). Moreover, the RDP3 program could detect the ‘NNYS09’ and ‘Trk7’ isolates as possible major and minor parental sequences, respectively, with 99.9% and 99.5% levels of confidence for both recombinants (data not shown). Positions 1 and 840 in each sequence were detected as the beginning and ending breakpoints for both isolates, respectively. Phylogenetic trees were constructed separately according to the non-recombination and detected recombination regions (data not shown). Both the ‘BORU93’ and ‘HORU94’ isolates were assigned as subgroup IA based on the non-recombination region (data not shown), while inclusion of the recombination region clustered these two isolates within subgroup II.

**Figure 3:**
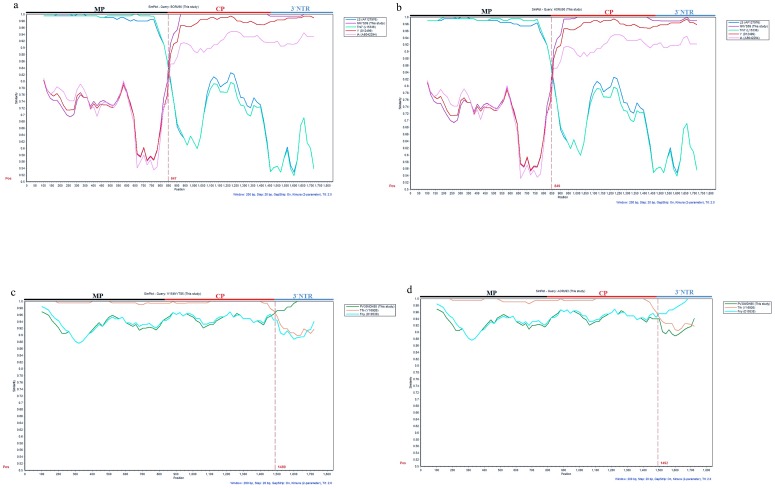
Phylogenetic relationship of CMV isolates on the basis of concatenation of nucleotide sequences of MP, CP and 3′NTR using Simplot. Four US CMV isolates, (a) BORU93, (b) HORU94, (c) V154NYT85, and (d) AORU93 were used as query sequences and six CMV isolates as reference sequences. Y-axis varies in identity percentage within a sliding window of 200 bp and a step size of 20 bp. Black vertical dashed line shows the proposed recombination break point. Sequences compared with the query sequence are indicated in the legend.

**Figure 4:**
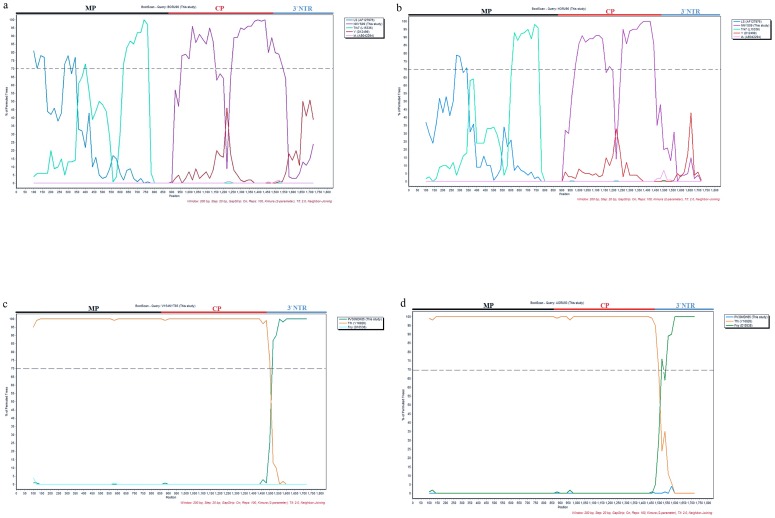
Bootscan analysis with recombinant CMV isolates as the query sequences. Query sequences included (a) BORU93, (b) HORU94, (c) V154NYT85, and (d) AORU93 which are illustrated on the upper portion of the figures. Sequences compared with the query sequences are indicated in the legend. Analysis was performed with a sliding window of 200 bp and a step size of 20 bp. The Y-axis illustrates the percentage of permuted trees in which each selected isolate clustered with the query sequence. The 70% cutoff level, representing possible recombination, is indicated by the dashed line.

To confirm the result obtained from the RDP3, phylogenetic and BootScan analyses using ‘BORU93’ and ‘HORU94’ isolates as the query sequences were performed with the Simplot package. We used the assumed parents estimated by the RDP3 (NNYS09 and Trk7) and representatives from both subgroups I and II (NNYS09, PNYC90, Fny and IA for subgroup I and Trk7, LS, and Q for subgroup II) as references. When the phylogenetic analysis was performed for ‘BORU93’, a recombination point was detected at position 825 (Figure 3a) of the sequence alignment, while for ‘HORU94’, this position was nucleotide 841 (Figure 3b). Both of these positions were close to position 844 estimated by the RDP3 program. To confirm the results obtained by Simplot, similar sequences were used for a bootscanning analysis. The basic principle of bootscanning is that ‘mosaicism’ is suggested when one observes high levels of phylogenetic relatedness between a query sequence and more than one reference sequence in different genomic regions [51]. Evidence of recombination is typically considered to be supported when 70% of permuted trees support a particular grouping of sequences. Bootscan analyses demonstrated that the ‘BORU93’ and ‘HORU94’ isolates were built from a movement protein region related to the isolate from subgroup II, and a CP and 3′NTR region related to the isolate from subgroup IA (Figure 4).

Furthermore, we obtained evidence for other recombination events between CMV subgroups IA and IB with select isolates. Phylogenetic trees placed isolates ‘V154NYT85’ and ‘AORU93’ in subgroup IB based on the MP and CP regions (data not shown), whereas they were clustered along with other subgroup IA isolates according to the 3′NTR region of RNA 3 (data not shown). These two recombination events were further confirmed by concatenating three corresponding sequences (MP, CP and the 3′NTR) and then scanning aligned sequences by using the RDP3 package with multiple methods. Six out of seven methods suggested recombination events for these two isolates with highly significant P-values (Tables S2). Moreover, the RDP3 program detected the ‘PV30MDH85’ and ‘Tfn’ isolates as possible major and minor parental sequences, respectively with high levels of confidence, 99 and 96%, respectively, for both recombinants (data not shown). Positions 46 and 1514 of the concatenated sequence for ‘V154NYT85’ and 26 and 1732 for ‘AORU93’ were detected as the beginning and ending breakpoints of the recombination events (Table S2). Phylogenetic and Bootscan analyses using isolates ‘V154NYT85’ and ‘AORU93’ as query sequences, assumed parents estimated by RDP3 and representatives from both subgroups IA and IB confirmed the results obtained by RDP3 (Figures 3 and 4).

We also examined sequence alignments of other genomic regions by RDP3 to detect any possible recombination event(s). Several isolates in each region were predicated to experience recombination events (data no shown), but less than four out of seven methods with non-expected, significant P-values supported them. Also, Bootscan analysis with the Simplot program did not confirm these isolates as candidate recombinants. Therefore, they were not considered as candidate recombinants in our analyses.

### Selection pressure on different coding regions

To determine direction of the selective constraints imposed on different genomic regions of CMV and to compare the degree of selection among coding regions, patterns of selection in these genes were further analyzed. For this, the ratio of non-synonymous and synonymous substitutions was estimated for each segment (Table 2). There was no evidence for positive selection in any of the CMV coding regions among isolates included in this investigation. The mean ω (dN/dS) values estimated from pairwise comparisons between sequences were less than 1 for all regions with the highest value observed for the 2b gene (Table 2). These ratios of ω indicated that CMV coding regions were subjected to negative or purifying selection (ω < 1). The 1a, MP and CP genes showed low ω (dN/dS) ratios, suggesting high selective pressure, whereas this ratio was almost 4 times greater for 2a and 2b (Table 2), suggesting a greater tolerance for amino acid substitutions among these latter two genes. To identify selection at individual codons in each coding region, we applied three complementary maximum- likelihood methods (SLAC, FEL and REL). Overall, the estimated ω (dN/dS) ratios using these methods further confirmed the result obtained from pairwise comparisons. Specifically, sites 72 (V → A, A → T, T → N or A→ V, T → M) in the 2b, 25 (P → L, P → S) in the CP, 76 (D → V, D → E) in the 2a, 258 and 270 (A → V,V → I, A → T, T → S, A → V) in the MP were accepted as positively selected sites by two methods (Table 3).

**Table 3:** Codon positions of the coding regions in the U.S. CMV isolates affected by positive selection.

Coding region	Site	SLAC dN-dS	SLAC P-value	FEL dN-dS	FEL P-value	REL dN-dS	Bayes Factor
2a	76**	5.710	0.400	51.346	**0.064**	1.381	**120.219**
2b	72**	7.466	0.167	28.203	**0.059**	9.7450	**6190.190**
MP	258**	4.448	0.446	10.315	**0.092**	0.740	**164.668**
	270**	6.681	0.296	15.978	**0.040**	0.784	**279.563**
CP	25**	8.110	0.296	23.719	**0.077**	0.703	**356.592**

**Sites detected as statistically significant by two methods.

Statistically significant values have been illustrated as bold.

## Discussion

Changes in genetic composition of a virus population in addition to new phenotypes which can arise as a result of genetic exchanges (e.g. reassortment and recombination), can compromise effectiveness of disease control strategies [61]. Therefore, an improved understanding of genetic structure and associated factors or selective forces driving CMV evolution can help to design improved disease management strategies. In the current study, our goal was to capture a snapshot of genetic diversity of CMV. Furthermore, we investigated sources of variation in genetic diversity observed among a select set of U.S. CMV isolates collected from different host plants and regions of the country. Overall, the U.S. CMV isolates included in this investigation exhibited low genetic diversity. The observed low genetic diversity among isolates in this investigation is not surprising, however, as similar results have previously been reported for populations of this virus in large geographic regions including California [27] and Spain [25], [29]. A founder effect has been suggested as a partial explanation potentially shaping observed genetic structure of CMV [27], [29]. Genetic bottleneck(s) may also have contributed to this observed low genetic diversity and function to minimize the extent of genetic variation. Genetic bottlenecks during CMV systemic movement in host plants and CMV transmission by the aphid vector(s) have previously been reported [62], [63]. This low genetic variation is true for most plant virus populations [22] and our finding is consistent with the concept that genetic stability is the rule in natural plant virus populations [22]. Among U.S. CMV coding regions in the current study, the 2b gene demonstrated more genetic variability (π = 0.058) when compared with other regions. This result was consistent with previous investigations by Lin et al. (2004) and Liu et al. (2009), demonstrating that the 2b gene possessed the greatest diversity in the CMV genome. All CMV isolates included in this investigation belonged to subgroup I. If we consider these isolates as a reasonable approximation of the U.S. CMV population, result of the current study suggests that subgroup I is of greater prevalence than subgroup II in in the U.S., and this observation is consistent with previous results [27], [32]. Within subgroup I, IA has a worldwide distribution, while most of the previously described subgroup IB strains were from Asia [20], although presence of this subgroup in other regions such as Italy [64], Greece [65] and Brazil [66] has been reported. In the United States, presence of two CMV subgroup IB strains isolated from pepper in 1990 in CA and one strain isolated from banana in 1995 in Hawaii was reported for the first time [20], [27] The current study detected several subgroup IB isolates among historic CMV isolates and phylogenetic analysis further revealed presence of this specific subgroup in the U.S. prior to previous reports. However, no CMV subgroup IB was detected among recently collected isolates sampled from a single host, snap bean, in regions affected by the recent virus outbreaks (unpublished data).

Reassortment in RNA viruses with segmented genomes has been shown for animal and plant viruses [19], [27], [54], [67]–[71]. Here, we report phylogenetic evidence for natural, inter-subgroup reassortment between subgroups IA and IB for five CMV isolates included in this study. This type of reassortment has been previously reported for CMV isolates [19], [25], [27]. Although, we only assessed part of the RNA 1 sequence, we believe that this sequence is sufficient to distinguish subgroups IA and IB from each other.

This study also detected two CMV isolates as natural recombinants from a subgroup II pattern for the MP gene and a subgroup IA pattern for CP gene. Recombination was confirmed by both phylogenetic and computational analyses designed to detect recombination events. In similar studies investigating natural CMV populations in Spain, approximately 17% of the sequenced isolates possessed evidence for recombination derived principally from the RNA 3 of CMV subgroup IA and IB, with MP CMV (IA)/CP CMV (IB) as the most prevalent type of recombinant [25]. To our knowledge, this is the first report of recombination between subgroup I and II CMV isolates in RNA 3 in natural populations of CMV. Phylogenetic and computational analyses also detected recombination events between subgroup IA and IB in RNA3 with the MP and CP (IB)/3′NTR (IA) pattern. To construct phylogenetic trees, however, we removed the sequences of these four recombinants from our data base because recombination can mislead the phylogenetic estimation procedures [72].

A low frequency of genetic exchange (reassortment and recombination) among CMV isolates assessed in this research agrees with the previous results obtained from analysis of the genetic structure of field population of CMV in Spain and California [25]–[27], [29] and illustrates that these events are counter- selected in CMV natural populations [73].

An analysis of natural selection showed that negative (or purifying) selection was the predominant evolutionary force operating upon all CMV coding regions. This type of selection imposed on CMV encoded proteins has been shown previously [27], [35], [74]. On average, the evolutionary constraints exerted on proteins 1a, MP and CP were larger than on 2b and 2a. This observation is in agreement with previous observation from California CMV populations [27] showing that the 2b and 2a proteins are more flexible with regard to amino acid substitutions and is also consistent with the idea that different coding regions of CMV are under different constraints. However, the ω (dN/dS) ratio was higher for 2a and 2b genes in our research when compared to the ω (dN/dS) values of California CMV isolates, likely because we had a diverse collection of CMV isolates from both subgroups IA and IB and different geographic locations throughout the US. Selection can be associated with various factors such as structural features of the virus, host plant and an arthropod vector. Garcia-Arenal et al. (2001) illustrated that negative selection predominates during evolution of plant viruses when the entire genome is assayed and that this purifying selection is principally due to the internal and external constraints [22]. In the case of CMV, almost all encoded proteins have direct or indirect interactions either with the host plant (1a, 2a, 2b, MP and CP) or the insect vector (CP). Moreover, CMV possesses a very broad host range and can be transmitted by a large number of aphid vectors, but there remains some degree of specificity for both transmission and infectivity by this virus [14], [75]–[78]. For CMV to effectively adapt to this level of host and vector variation, we expected to see some degree of positive (diversifying) selection in portions of the CMV coding regions similar to those presented for other plant viruses in the past surveys [74], [79]–[82]. In the CP gene, we noted that site 25 was accepted as the positively selected codon in subgroup IA. This result corroborated previous reports illustrating diversifying selection for this codon [74]. This amino acid is located in the folded portion of CP [83] and it affects CMV transmission by aphids [14]. Hence, a positive selection pattern in this CP region could be related to the role of different aphid species in selection of different virus variants. Furthermore, site 76 in the 2a protein was shown to be under positive selection in the current study. These changes were detected in only a portion of subgroup IB isolates. Referencing *Brome mosaic virus* (BMV), another member of the family *Bromoviridae*, the N-terminal, 115 amino acids of the 2a protein are necessary to interact with the helicase domain of 1a protein [84], [85]. Accordingly, the detected positively selected site in the CMV 2a protein is probably involved in this interaction and is necessary for replication. Positively selected site 72 in the 2b protein located in the overlapping region with ORF2a was strongly accepted by all three maximum-likelihood methods, demonstrating a strong diversifying selection on this particular codon. Here again, 2b is a multifunctional protein involved in virus, long- distance movement, symptom induction, silencing suppressor and as a pathogenicity determinant [4], [6]–[8], [86]. Therefore, there is likely a high level of interaction between this protein and host components and, at least in the case of long- distance movement, this interaction may regulate host specificity [8]. This host-specific function and the extremely wide host range of CMV allow 2b to be considerably more tolerant to nucleotide and amino acid changes (highest estimated ω value among all coding regions). Multiple amino acid substitutions at this particular site suggest a greater selection potential for adaptation. However, no specific amino acid/host relationship was recognized for this particular site in this study. Instead, we found an amino acid/subgroup relationship in one of those substitutions in the 2b protein so that T → N occurred in only subgroup IB. Compared to the known domains of the CMV 2b protein with known functions, codon 72 does not belong to any [86], [87] previously described. Therefore, we have insufficient information at the current time to propose reasons for this selection. However, we hypothesize that amino acids located in this region of the protein are involved in an important function, because CMV isolates from California showed a greater average number of non-synonymous mutations than synonymous in a short region of the 2b gene (codons 81–93) [27]. Therefore, an improved understanding of the function of this region remains as an interesting aspect that warrants further investigation.

The selection determination methods applied in the current study also detected two positively selected sites 258 and 270 in the MP protein which were not reported in the previous study [74]. To our knowledge, there is no information about the function (s) of these mentioned codons.

Although, positive selections were detected at several sites in the 1a protein in the current study we did not accept them because only one out of three methods supported these selections. Most of these sites have been reported as the positively selected codons in previous studies [74]. One possible reason is that we only partially sequenced ORF1a. Therefore, if we had assessed the whole gene, perhaps we would have detected more positive codons with more methods and a higher level of statistical confidence.

It is also noteworthy to mention here that positively selected codons reported for the 2a and 2b proteins in this research, have not been reported in previous investigations [74] and may be unique. However, the number of positively selected sites for the CP among the range of CMV isolates included in this investigation was much less. There are possible explanations for these differences. First of all, different methods for detecting positive selection, may well account for the observed differences between these two studies. Secondly, we selected isolates from natural populations of CMV in our research composed of subgroups IA and IB in the US, while the previous research included only available CMV sequences in GenBank belonging to subgroups IA, IB and II obtained from both experimental and natural conditions with undefined constraints imposed upon the coding regions.

## Supporting Information

Table S1 (PDF)

Table S2 (PDF)
