# Supplementary material for: Genetic Structure and Molecular Variability of Cucumber mosaic virus Isolates in the United States
Source: PLoS One. 2014 May 6;9(5):e96582. doi: 10.1371/journal.pone.0096582 (PMC4012352; doi:10.1371/journal.pone.0096582)
Supplement: Table S2 — (PDF) [file pone.0096582.s002.pdf]

Supporting information

Table S2. Probability estimates for the US CMV isolates to be considered as recombinants.

Recombinant isolate	P-value for the six detection methods						Region (nt position)	Parental sequence	
	GENECONV	BootScan	MaxChi	Chimaera	SisCan	3SEQ		Major	Minor
BORU93	1.01E-51	7.49E-58	5.86E-31	3.76E-31	1.40E-31	7.13E-07	3-841	NNYS09	Trk7
HORU93	1.2E-50	7.32E-59	5.92E-31	3.50E-30	1.46E-31	7.23E-08	3-841	NNYS09	Trk7
V154NYT85	1.03E-2	2.16E-4	3.93E-5	3.53E-6	2.16E-19	4.69E-11	46-1514	PV30MDH85	Tfn
AORU93	1.03E-2	2.16E-4	3.93E-5	3.53E-6	2.16E-19	4.69E-11	46-1514	PV30MDH85	Tfn

Table S2. Accession numbers and country of origin of reference isolates and out-groups.

Strain	Accession numbers			Origin	Subgroup
	RNA1	RNA2	RNA3		
Fny	D00356	D00355	D10538	United States	IA
Y	D12537	D12538	D12499	Japan	IA
O	-	D10209	D00385	Japan	IA
Mf	AJ276479	AJ276480	AJ276481	South Korea	IA
Leg	D16403	D16406	D16405	Japan	IA
Nt9	D28778	D28779	D28780	Taiwan	IB
Tfn	Y16924	Y16925	Y16926	Italy	IB
IA	AB042292	AB042293	AB042294	Indonesia	IB
Ix	U20220	U20218	U20219	Philippines	IB
SD	AF071551	D86330	AB008777	China	IB
Q	X02733	X00985	M21464	Australia	II
LS	AF416899	AF416900	AF127976	United States	II
LY	AF198101	AF198102	AF198103	Australia	II
Trk7	AJ007933	AJ007934	L15336	Hungary	II
S	Y10884	Y10885	U37227	South Africa	II
2AIIL	-	-	AJ271416	United States	IB
OHW	-	-	U31220	United States	IB
HW2	-	-	U31219	United States	IB
PSV	U15728	U15729	U15730	United States	-
TAV	D10044	D10663	AJ277268	Australia	-
